# An efficient logarithmic estimator in stratified random sampling using single auxiliary variable

**DOI:** 10.1038/s41598-026-41448-9

**Published:** 2026-02-26

**Authors:** Fazal Shakoor, Muhammad Asif, Muhammad Atif, Khazan Sher

**Affiliations:** 1 Department of Statistics, University of Peshawar, Peshawar, Pakistan; 2 Department of Mathematics and Statistics, College of Science, Imam Mohammad Ibn Saud Islamic University (IMSIU), Riyadh, Saudi Arabia; 3 Department of Statistics, Government Hakeem Abdul Jalil Nadvi Degree College Gulbahar Peshawar, Peshawar, Pakistan

**Keywords:** Auxiliary, Bias, Efficiency, Logarithmic estimator, MSE, PRE, Ratio estimator, Stratified random sampling, Engineering, Mathematics and computing

## Abstract

This paper proposes a novel logarithmic-type estimator for the estimation of the population mean under stratified random sampling when a single auxiliary variable is available. By employing a logarithmic transformation, the suggested estimator more effectively exploits auxiliary information, leading to improved estimation accuracy. Analytical expressions for the bias and precision of the estimator are derived, and theoretical conditions are established under which the proposed estimator dominates several existing estimators. The empirical performance is assessed using three real-life datasets, along with a simulation study based on three artificial populations of size 1000 generated from a normal distribution. Samples of sizes *n* = 50, 100, and 150 are drawn to evaluate estimator behavior under varying sampling intensities. The findings consistently show that the proposed estimator attains higher percentage relative efficiency and superior precision compared to competing estimators across both real and simulated datasets. These results demonstrate that the estimator offers substantial gains in survey accuracy, particularly when a strong association exists between the study and auxiliary variables.

**Supplementary Information:**

The online version contains supplementary material available at 10.1038/s41598-026-41448-9.

## Introduction

The issue of bias versus unbiasedness has remained a fundamental consideration in statistical estimation. Although unbiased estimators are theoretically appealing, they are not always the most effective choice in practical applications, especially in situations characterized by limited variability. In many applied settings, biased estimators are preferred because they tend to produce estimates that are closer to the true population parameter on average. Despite introducing a certain degree of bias, such estimators often exhibit improved performance when assessed through precision-based criteria, since a reduction in variability may compensate for the presence of bias.

As sample information increases, estimators that permit a modest amount of bias while achieving greater precision become particularly attractive. This reflects the well-known bias–precision trade-off, where a controlled increase in bias can lead to a substantial decrease in variability, thereby enhancing overall estimator reliability. In investigation sampling, the actual usage of supplementary information that displays a solid association with the study flexible has developed a widely adopted approach. The integration of such auxiliary variables significantly improves estimator accuracy and efficiency, playing a crucial role at both the design and estimate periods of the sampling process.

The effective choice of auxiliary variables plays a crucial role in dropping the estimation error connected with population parameters. When appropriately selected, such variables can lead to a substantial improvement in estimator precision. Among the commonly used approaches, ratio-type estimators have attracted substantial attention because of their capacity toward take advantage of on the functional association among the study and auxiliary variables. These estimators are mostly advantageous for approximating population means and wholes, frequently delivering more accuracy than conventional estimators. Many times, numerous investigators have contributed to this area by proposing ratio and regression-based estimators employing a wide range of functional transformations, thereby considerably advancing the theory and practice of survey sampling^1^. Inside the context of SRS selection, some authors have developed estimators based on mixed ratio-type structures. Koyuncu et al.^2^ examined the estimators originally proposed in^3^, demonstrating their effectiveness under the stratified sampling framework. Subsequently, Koyuncu et al.^4^ introduced an integrated version of the estimator proposed by^5^, which resulted in further gains in estimation efficiency. Singh et al.^6^ extended this line of research by proposing a broad class of estimators that utilize auxiliary information within the simple random sampling design, while Singh et al.^7^ later introduced a highly efficient family of estimators under the same framework. A comprehensive overview of these developments, together with the associated literature, is presented in^8^. More recently, Shakoor et al.^23^ proposed a logarithmic ratio-type estimator under simple random sampling, which serves as the basis for the present extension to the stratified random sampling setting.

Stratified sampling methods that incorporate auxiliary information have been extensively applied diagonally an extensive choice of technical and applied corrections, including physical science, manufacturing, and ecological disciplines. In physical science, such approaches facilitate more precise estimation of particle densities in high-energy experiments, astrophysical parameters, and substantial properties^9^. The principal aim of the present study, within the stratified random sampling framework, is to progress and assess well-organized estimators of the population mean using a single auxiliary variable. To this end, two new classes of estimators for finite populations are proposed. Their statistical properties are rigorously examined through the derivation of bias and precision measures under first-order approximation, offering a clear evaluation of their efficiency and comparative performance.

Ongoing developments in sampling theory continue to emphasize the need for improved estimation procedures that deliver greater statistical accuracy. The estimator introduced in this study contributes to this objective by providing a more efficient framework for population mean estimation. By strategically incorporating auxiliary information within each stratum, the proposed estimator enhances both accuracy and reliability relative to existing approaches, thereby making more effective use of available information.

The estimation of the finite population mean remains a fundamental problem in stratified random sampling. Conventional estimators, including the stratified sample mean, ratio estimator, and regression estimator, are widely applied but often depend on restrictive conventions and might not entirely utilize the association among the study and supplementary variables. Even if additional present methods such as generalized regression and exponential ratio estimators offer improvements, they still exhibit certain limitations. To overcome these shortcomings, this study introduces a new class of estimators that better exploit auxiliary information within the stratified sampling design. These estimators are particularly well-suited to situations involving nonlinear relationships or departures from normality, where traditional estimators may lose efficiency. Evidence from simulation experiments and empirical applications demonstrates the superior performance of the proposed estimators, confirming their practical relevance and effectiveness for both researchers and practitioners.

## Novelty and significance

The study suggests a logarithmic ratio-type estimator for estimating the population mean within the Simple Random Sampling (SRS) framework, particularly in situations where a strong-correlation exists among the study and supplementary variables. The novelty of the proposed estimator lies in its structural formulation and application within the stratified random sampling framework, which fundamentally distinguishes it from existing logarithmic and ratio-type estimators. Specifically, unlike traditional ratio and logarithmic estimators that primarily rely on linear or direct transformations of auxiliary information, the proposed estimator introduces a modified logarithmic functional form that is systematically incorporated at the stratum level and then aggregated across strata. This formulation allows the estimator to more efficiently utilize auxiliary information by reducing variability arising from heterogeneous strata, which is not explicitly addressed in existing estimators. First-order approximations of the bias and Mean Square Error (MSE) of the proposed estimator are derived under stratified random sampling. Its efficiency is assessed numerically using real datasets, while finite-sample properties are further investigated through Monte Carlo simulations designed to reflect realistic engineering sampling conditions, including heterogeneous stratum sizes.

## Methodology

Let a finite population$\:\:\varOmega\:={\{\varOmega}_{1},\:{\varOmega}_{1},\dots\:{\varOmega}_{N}$} taking N different and recognizable components divided and obsessed by K strata. And let y be the study and x be the auxiliary variables taking values from $\:{y}_{hi}$ and $\:{x}_{hi}$, respectively, for *i*th units where $\:\left(i=\mathrm{1,2},\dots\:N\right)$ in *h*^th^ stratum consisting of $\:{N}_{h}$ units such that $\:\sum\:_{h=1}^{K}{N}_{h}=N$. And let $\:{n}_{h}$ be the sample size drawn from *h*^th^ stratum by using simple random sampling without replacement methods such that $\:\sum\:_{h=1}^{K}{n}_{h}=n$. And Let $\:{\bar{y}}_{str}=\sum\:_{h=1}^{K}{W}_{h}{\bar{y}}_{h}$, where $\:{\bar{y}}_{h}=\:\frac{1}{{n}_{h}}\sum\:_{i=1}^{{n}_{h}}{y}_{hi}$. And let $\:{\mu}_{y}=\sum\:_{h=1}^{K}{W}_{h}{\bar{Y}}_{h}$, where $\:{\bar{Y}}_{h}=\:\frac{1}{{N}_{h}}\sum\:_{i=1}^{{N}_{h}}{y}_{hi}$ and $\:{W}_{h}=\frac{{N}_{h}}{N}$ is the stratum weight. Also the expression we can write for the variable x. let $\:{s}_{yh}^{2}=\frac{1}{{n}_{h-1}}\sum\:_{i=1}^{{n}_{h}}{\left({y}_{hi}-{\bar{Y}}_{h}\right)}^{2}$ and $\:{s}_{xh}^{2}=\frac{1}{{n}_{h-1}}\sum\:_{i=1}^{{n}_{h}}{\left({x}_{hi}-{\bar{x}}_{h}\right)}^{2}$ be the sample variance of y and x respectively, in the *h*^th^ stratum corresponding to the population variance $\:{S}_{yh}^{2}=\frac{1}{{N}_{h-1}}\sum\:_{i=1}^{{N}_{h}}{\left({y}_{hi}-{\mu}_{yh}\right)}^{2}$ and $\:{S}_{xh}^{2}=\frac{1}{{N}_{h-1}}\sum\:_{i=1}^{{N}_{h}}{\left({x}_{hi}-{\mu}_{xh}\right)}^{2}$.

Some of the properties to obtain bias and MSE of the estimators, which are define in the following terms.

Let suppose that $\:{\bar{y}}_{str}=\sum\:_{h=1}^{K}{W}_{h}{\bar{y}}_{h}=\:{\mu}_{y}\left(1+{\in}_{0}\right)$ and similarly, $\:{\bar{x}}_{str}=\sum\:_{h=1}^{K}W{\bar{x}}_{h}=\:{\mu}_{x}\left(1+{\in}_{1}\right)$ These are the complete means of the study variable y and the supplementary variable x, obtained through SRS, are represented by $\:{\bar{y}}_{str}$​ and $\:{\bar{x}}_{str}$​, correspondingly. These comparative errors are classically trivial, with expected values near zero under standard assumptions, and possess well-defined variances and covariance. Such properties are essential in examining the behavior of estimators that incorporate secondary evidence, including ratio-type and regression-type estimators.

$$\:E\left({\in}_{0}\right)=E\left({\in}_{1}\right)=0$$

$$\:E\left({\in}_{0}^{2}\right)={C}_{y}^{2}=\sum\:_{h=1}^{K}{W}_{h}{\pi}_{h}{C}_{yh}^{2}={\nu}_{40}$$

$$\mathrm{let}\:E\left({\in}_{1}^{2}\right)={C}_{x}^{2}=\sum\:_{h=1}^{K}{W}_{h}{\pi}_{h}{C}_{xh}^{2}={\nu}_{04}$$

and

$$\:E\left({\in}_{0}{\in}_{1}\right)=\sum\:_{h=1}^{K}{W}_{h}^{2}{\pi}_{h}{\rho}_{yxh}{C}_{yh}{C}_{xh}={\nu}_{44}$$

Where, $\:{C}_{yh}^{2}=\frac{{S}_{yh}^{2}}{{\bar{Y}}^{2}}$ and $\:{C}_{xh}^{2}=\frac{{S}_{xh}^{2}}{{\bar{X}}^{2}}$ are population coefficient of variations of the study and auxiliary variables, respectively. Similarly, $\:{\pi}_{h}=\frac{1}{{n}_{h}}-\frac{1}{{N}_{h}}$, is the finite population correction factor, and $\:{W}_{h}=\frac{{N}_{h}}{N}$ is the stratum weight, and $\:{R}_{xy}=\frac{{\mu}_{x}}{{\mu}_{y}}$.

## Existing estimators

In the situation of SRS with a single supplementary variable, several estimators been proposed to improve the estimate of the fixed population mean. Researchers have explored a variety of techniques that utilize the available auxiliary information to produce more precise and efficient estimates^4^. These approaches are specifically designed to account for the stratified nature of the population. The commonly used estimator in this setting is the unbiased estimator of the population mean, which serves as a standard reference and is expressed mathematically as follows:

1 $$\:{T}_{1}=\sum\:_{h=1}^{K}{W}_{h}{\bar{y}}_{h}$$

The variance associated with the classical unbiased estimator is defined by the following expression:

2 $$\:MSE\left({T}_{1}\right)={\bar{Y}}^{2}{\nu}_{40}$$

Although the classical estimator is unbiased, it may suffer from relatively large variance. To mitigate this limitation, the availability of auxiliary information $\:{X}_{i}$that is correlated with the study variable $\:{Y}_{i}$ motivates the use of the traditional ratio estimator, as suggested in^10^, which is defined as follows:

3 $$\:{T}_{2}={\bar{y}}_{str}\frac{{\mu}_{x}}{{\bar{x}}_{str}}$$

The analytical expressions used for the bias and MSE corresponding to Cochran’s ratio-estimator are presented below:

4 $$\:Bias\left({T}_{2}\right)=\bar{Y}\left({\nu}_{04}-{\nu}_{44}\right)$$

5 $$\:MSE\left({T}_{2}\right)={\bar{Y}}^{2}\left({\nu}_{40}+{\nu}_{04}-2{\nu}_{44}\right)$$

An exponential ratio-type estimator was introduced by Bahl et al.^11^. Its mathematical form, along with the corresponding bias and MSE expressions, is given as follows:

6 $$\:{T}_{3}={\bar{y}}_{str}exp\left[\frac{{\mu}_{x}-{\bar{x}}_{str}}{{\mu}_{x}+{\bar{x}}_{str}}\right]$$

7 $$\:Bias\left({T}_{3}\right)=\bar{Y}\left(\frac{3}{8}{\nu}_{04}-\frac{1}{2}{\nu}_{44}\right)$$

8 $$\:MSE\left({T}_{3}\right)={\bar{Y}}^{2}\left({\nu}_{40}+\frac{{\nu}_{04}}{4}-2{\nu}_{44}\right)$$

Drawing on the studies conducted in^12,13^, a ratio estimator has been proposed under the assumption that the population coefficient of is identified.

9 $$\:{T}_{4}={\bar{y}}_{str}\left(\frac{{\mu}_{x}+{C}_{x}}{{\bar{x}}_{str}+{C}_{x}}\right)$$

Using first-order approximation techniques, the estimators are derived, and their associated bias and MSE are discussed in the following:

10 $$\:Bias\left({T}_{4}\right)=\bar{Y}\left({\vartheta\:}^{2}{\nu}_{04}-\vartheta\:{\nu}_{44}\right)$$

11 $$\:MSE\left({T}_{4}\right)={\bar{Y}}^{2}\left({\nu}_{40}+{\vartheta\:}^{2}{\nu}_{04}-2\vartheta\:{\nu}_{44}\right)$$

In the above Eq. (10) and Eq. (11),

$$\:\vartheta\:=\sum\:_{h=1}^{K}{W}_{h}\frac{{\mu}_{xh}}{\left({\mu}_{x}+{C}_{x}\right)}$$

Upadhyaya et al.^14^ extended the estimator proposed in Sisodia & Dwivedi by including the product of the supplementary variable mean and its kurtosis quantity. The structure of the modified estimator, together with expressions for its bias and MSE, is provided in Eq. (12).

12 $$\:{T}_{5}={\bar{y}}_{str}\sum\:_{h=1}^{K}{W}_{h}\left(\frac{{\mu}_{xh}{\beta}_{2h}\left(x\right)+{C}_{xh}}{{\mu}_{xh}{\beta}_{2h}\left(x\right)+{C}_{xh}}\right)$$

The Bias and MSE of the suggested estimator are as follows:

13 $$\:Bias\left({T}_{5}\right)=\bar{Y}\left(\phi\:{\nu}_{40}-{\nu}_{44}\right)$$

14 $$\:MSE\left({T}_{5}\right)={\bar{Y}}^{2}\left({\nu}_{40}+{\phi\:}^{2}{\nu}_{04}-2\phi\:{\nu}_{44}\right)$$

In the above Eq. (13) and Eq. (14) here $\:\phi\:=\frac{{\mu}_{xh}{\beta}_{2h}\left(x\right)}{{\mu}_{xh}{\beta}_{2h}\left(x\right)+{C}_{xh}}$.

Building on the approach of Chaudhary et al.^15^., a general class of population mean estimators was developed by Kadilar and Cingi^16^, as described below:

15 $$\:{T}_{6}={\bar{y}}_{str}{\left[\frac{a{\mu}_{x}+b}{\alpha\:\left(a{\bar{x}}_{str}+b\right)+\left(1-\alpha\:\right)\left(a{\mu}_{x}+b\right)}\right]}^{\tau\:}$$

By assigning specific values to the factors (a, b,τ) = 0,1, and − 1, and $\:\alpha\:$ a variety of estimators can be generated. The corresponding bias and MSE of the estimator are given as follows:

16 $$\:Bias\left({T}_{6}\right)=\bar{Y}\left(\frac{\tau\:\left(\tau\:+1\right)}{2}{\alpha\:}^{2}{\theta\:}^{2}{\nu}_{04}-\alpha\:\tau\:\theta\:{\nu}_{44}\right)$$

17 $$\:MSE\left({T}_{6}\right)={\bar{Y}}^{2}\left({\nu}_{40}+{\alpha\:}^{2}{\tau\:}^{2}{\theta\:}^{2}{\nu}_{04}-2\alpha\:\tau\:\theta\:{\nu}_{44}\right)$$

Where,

18 $$\:\theta\:=\frac{a{\mu}_{x}}{a{\mu}_{x+b}},\:\alpha\:=\frac{{\nu}_{44}}{\theta\:\tau\:{\nu}_{04}}.$$

Following the studies in^17,18^, a class of exponential estimators for population mean estimation under simple random sampling without replacement was introduced.

19 $$\:{T}_{7}={\bar{y}}_{str}\left[{\alpha}_{1}exp\left\{\frac{a\left({\mu}_{x}-{\bar{x}}_{str}\right)}{a\left({\bar{x}}_{str}+{\mu}_{x}\right)+2b}\right\}+{\alpha}_{2}exp\left\{\frac{a\left({\bar{x}}_{str}-{\mu}_{x}\right)}{a\left({\bar{x}}_{str}+{\mu}_{x}\right)+2b}\right\}\right]$$

The above estimator MSE is gained as:

20 $$\:{MSE(T}_{7})=\frac{{\nu}_{01}{\nu}_{02}-{\nu}_{22}^{2}}{{\nu}_{01}+{\nu}_{02}-2{\nu}_{22}}$$

where,

$$\:{\nu}_{01}=\sum\:_{h=1}^{K}{W}_{h}^{2}{\pi}_{h}{\bar{Y}}_{h}^{2}\left[{C}_{yh}^{2}+\frac{1}{4}\vartheta\:{C}_{xh}^{2}\left(\vartheta\:-4K\right)\right]$$

$$\:{\nu}_{02}=\sum\:_{h=1}^{K}{W}_{h}^{2}{\pi}_{h}{\bar{Y}}_{h}^{2}\left[{C}_{yh}^{2}+\frac{1}{4}\vartheta\:{C}_{xh}^{2}\left(\vartheta\:+4K\right)\right]$$

$$\:{\nu}_{22}=\sum\:_{h=1}^{K}{W}_{h}^{2}{\pi}_{h}{\bar{Y}}_{h}^{2}\left[{C}_{yh}^{2}-\frac{1}{4}{\vartheta\:}^{2}{C}_{xh}^{2}\right]$$

$$\:\vartheta\:=\frac{a{\mu}_{x}}{a{\mu}_{x}+b},\:and\:K=\frac{{\rho}_{h}{C}_{yh}}{{C}_{xh}}.$$

Similarly, the ratio-cum-exponential type estimator proposed by Izunobi and Onyeka^19^ is presented in Eq. (21) as follows:

21 $$\:{T}_{8}={\bar{y}}_{str}{\left[\frac{{\bar{x}}_{str}}{{{\mu}_{x}}_{str}}\right]}^{{a}_{2}}exp\left[\frac{{{\mu}_{x}}_{str}-{\bar{x}}_{str}}{{{\mu}_{x}}_{str}+{\bar{x}}_{str}}\right]$$

Where, $\:{\bar{x}}_{str}=\sum\:_{i=1}^{K}{W}_{h}\left({k}_{h}{\bar{x}}_{h}+{p}_{h}\right)$ and $\:{{\mu}_{x}}_{str}=\sum\:_{i=1}^{K}{W}_{h}\left({k}_{h}{{\mu}_{x}}_{h}+{p}_{h}\right)$ Here, $\:{k}_{h}$and $\:{p}_{h}$​ are functions of known parameters of the auxiliary variable, such as its coefficient of kurtosis and coefficient of variation. The bias and MSE of the proposed estimator are presented below.

22 $$\:Bias\left({T}_{8}\right)=\:\bar{Y}\left[\frac{1}{2}\left\{{\tau}_{2}\left({\tau}_{2}-1\right)-{\phi\:}^{2}-2{\tau}_{2}\phi\:\right\}{\nu}_{02}+\left({\tau}_{2}+\phi\:\right){\nu}_{44}\right]$$

23 $$\:MSE\left({T}_{8}\right)=\sum\:_{h=1}^{K}{{W}_{h}}^{2}{\vartheta}_{h}\left({S}_{yh}^{2}+{\left({\tau}_{2}-\phi\:\right)}^{2}{S}_{yh}^{2}+2\left({\tau}_{2}-\phi\:\right)R{S}_{yxh}\right)$$

$\:\phi\:=\frac{a{\mu}_{x}}{2\left(a{\mu}_{x}+p\right)}$ and $\:{\tau}_{2}$ are the minimizing constant.

24 $$\:MSE\left({T}_{8}\right)=\bar{Y}{\nu}_{40}\left(1-{\rho}_{t}\right)$$

The influence $\:{\rho}_{t}$ denotes the overall association factor across all strata and is well-defined as follows:

$$\:{{\rho}_{t}}^{2}=\frac{{\left(\sum\:_{h=1}^{K}{{W}_{h}}^{2}{\vartheta}_{h}{\rho}_{h}{S}_{yh}{S}_{xh}\right)}^{2}}{\sum\:_{h=1}^{K}{{W}_{h}}^{2}{\vartheta}_{h}{S}_{xh}^{2}\sum\:_{h=1}^{K}{{W}_{h}}^{2}{\vartheta}_{h}{S}_{yh}^{2}}$$

Motivated by Izunobi and Onyeka^19^, a new natural logarithmic ratio-type estimator is proposed to improve estimation of the population mean $\:{\mu}_{y}$​. Denoted by $\:\left({\mu}_{x}\right)$​, the estimator is constructed by taking the ratio of the sample mean of y to the natural logarithm of the sample mean of the auxiliary variable, and then multiplying by the natural logarithm of the known population mean of the auxiliary variable. The estimator is developed within the framework of simple random sampling without replacement (SRSWOR).

## Proposed Ln-type estimators

Motivated by the work of Izunobi and Onyeka^19^ and Shakoor et al.^23^, we propose a logarithmic ratio type estimator for the population mean under Stratified RSWOR. The estimator is constructed by means of the natural logarithm of the identified population mean of the secondary variable, $\:{T}_{Ln\left(str\right)}$​, and is defined as follows:

25 $$\:{T}_{Ln\left({\mu}_{xstr}\right)}=\left({K}_{1}{\bar{y}}_{str}+{K}_{2}\right)\left(\frac{{\mu}_{xstr}}{{\bar{x}}_{str}-{\mu}_{xstr}}\right)Ln\left(\frac{{\bar{x}}_{str}}{{\mu}_{xstr}}\right)$$

Given that $\:Ln\left(\frac{{\bar{x}}_{str}}{{\mu}_{xstr}}\right)>0$, imposing this constraint guarantees the validity of the logarithmic transformation and, consequently, ensures that the estimator is defined over an admissible range of values.

The proposed logarithmic ratio-type estimator, $\:{T}_{Ln\left(str\right)}$​, is constructed by distributing the SRS of the study variable $\:\left({\bar{y}}_{str}\right)$, by the natural logarithm of the stratified sample mean of the supplementary variable, $\:\left({\bar{x}}_{str}\right)$ and then multiplying the result by the natural logarithm of the known population mean of the secondary variable, $\:Ln\left({\mu}_{xstr}\right)$.

To proof the proposed estimator in Eq. (25) let,

$$\:{e}_{0}=\frac{{\bar{y}}_{str}-{\mu}_{y}}{{\mu}_{y}}$$

$$\:{e}_{1}=\frac{{\bar{x}}_{str}-{\mu}_{x}}{{\mu}_{x}}$$

then

26 $$\:E\left({e}_{0}\right)\:=\:E\left({e}_{1}\right)=0$$

$$\:E\left({\in}_{0}^{2}\right)={C}_{y}^{2}=\sum\:_{h=1}^{K}{W}_{h}{\pi}_{h}{C}_{yh}^{2}={\nu}_{40}$$

27 $$\:\:E\left({\in}_{1}^{2}\right)={C}_{x}^{2}=\sum\:_{h=1}^{K}{W}_{h}{\pi}_{h}{C}_{xh}^{2}={\nu}_{04}$$

and

28 $$\:E\left({\in}_{0}{\in}_{1}\right)=\sum\:_{h=1}^{K}{W}_{h}^{2}{\pi}_{h}{\rho}_{yxh}{C}_{yh}{C}_{xh}={\nu}_{44}$$

Where, $\:{C}_{yh}^{2}=\frac{{S}_{yh}^{2}}{{\bar{Y}}^{2}}$ and $\:{C}_{xh}^{2}=\frac{{S}_{xh}^{2}}{{\bar{X}}^{2}}$ are population coefficient of variations of the study and auxiliary variables, respectively. Furthermore, $\:{\pi}_{h}=\frac{1}{{n}_{h}}-\frac{1}{{N}_{h}}$, is the finite population correction factor, and $\:{W}_{h}=\frac{{N}_{h}}{N}$ is the stratum weight, and $\:{R}_{xy}=\frac{{\mu}_{x}}{{\mu}_{y}}$.

Then the above Eq. (25) can be expressed in relationships of sampling errors as follows in command on the way to get the estimator’s MSE.

29 $$\:{T}_{Ln\left(str\right)}=\left({k}_{1}{\bar{Y}}_{str}\left(1+{e}_{0}\right)+{k}_{2}\right)\left(\frac{{\mu}_{xstr}}{{{\mu}_{xstr}}_{{e}_{1}}}\right)Ln\left(1+{e}_{0}\right)$$

To expand the proposed estimator up the first order of approximation and apply the logarithmic Series and ignoring the high order terms. These expressions can be written is in Eq. (30) as:

30 $$\:{T}_{Ln\left(str\right)}=\:\left({k}_{1}{\bar{Y}}_{str}+{k}_{1}{{\bar{Y}}_{str}e}_{0}+{k}_{2}\right)\left(1-\frac{{e}_{1}}{2}+\frac{{e}_{1}^{2}}{3}\dots\:\right)$$

Similarly, when multiplying both terms in Eq. (30) the following equation we can write in Eq. (31) as:

31 $$\:\left[{T}_{Ln\left(str\right)}-\:{\bar{Y}}_{str}\right]={k}_{1}{\bar{Y}}_{str}-{\bar{Y}}_{str}-\frac{{k}_{1}{\bar{Y}}_{str}{e}_{1}}{2}+\frac{{k}_{1}{\bar{Y}}_{str}{e}_{1}^{2}}{3}+{k}_{1}{\bar{Y}}_{str}{e}_{0}-\frac{{k}_{1}{\bar{Y}}_{str}{e}_{0}{e}_{1}}{2}+{k}_{2}-\frac{{k}_{2}{e}_{1}}{2}+\frac{{k}_{2}{e}_{1}^{2}}{3}$$

Squaring both side of Eq. (31),

32 $$\begin{aligned}{\left[{T}_{Ln\left(str\right)}-\:{\bar{Y}}_{str}\right]}^{2}&=\:{\bar{Y}}_{str}^{2}+{k}_{1}^{2}{\bar{Y}}_{str}^{2}+\frac{{k}_{1}^{2}{\bar{Y}}_{str}^{2}{e}_{1}^{2}}{4}+{k}_{1}^{2}{\bar{Y}}_{str}^{2}{e}_{0}^{2}+{k}_{2}^{2}+\frac{{k}_{2}^{2}{e}_{1}^{2}}{4}-2{k}_{1}{\bar{Y}}_{str}^{2}-{k}_{1}^{2}{\bar{Y}}_{str}^{2}{e}_{1} +\frac{{2k}_{1}^{2}{\bar{Y}}_{str}^{2}{e}_{1}^{2}}{3}+{k}_{1}^{2}{\bar{Y}}_{str}^{2}{e}_{0}-{k}_{1}^{2}{\bar{Y}}_{str}^{2}{e}_{0}{e}_{1}\\ & \quad+{2k}_{1}{k}_{2}{\bar{Y}}_{str}-{k}_{1}{k}_{2}{\bar{Y}}_{str}{e}_{1} +\frac{{2k}_{1}{\bar{Y}}_{str}{e}_{1}^{2}}{3}+{k}_{1}{\bar{Y}}_{str}^{2}{e}_{1}-\frac{{2k}_{1}{\bar{Y}}_{str}^{2}{e}_{1}^{2}}{3}-2{k}_{1}{\bar{Y}}_{str}^{2}{e}_{0}+{k}_{1}{\bar{Y}}_{str}^{2}{e}_{0}{e}_{1}-2{k}_{2}{\bar{Y}}_{str}+{k}_{2}\bar{Y}{e}_{1}-\frac{{2k}_{2}\bar{Y}{e}_{1}^{2}}{3}\\ & \quad-\frac{{k}_{1}^{2}\bar{Y}{e}_{1}^{2}}{3}-{k}_{1}^{2}{\bar{Y}}_{str}^{2}{e}_{0}{e}_{1}-{k}_{1}{k}_{2}{\bar{Y}}_{str}{e}_{1}+{k}_{1}{k}_{2}{\bar{Y}}_{str}{e}_{1}^{2}+\frac{{2k}_{1}{k}_{2}{\bar{Y}}_{str}{e}_{1}^{2}}{3}+{2k}_{1}{k}_{2}{\bar{Y}}_{str}{e}_{0}-{2k}_{1}{k}_{2}{\bar{Y}}_{str}{e}_{0}{e}_{1}-{k}_{2}^{2}{e}_{1}+\frac{2{k}_{2}^{2}}{3}\end{aligned}$$

Now by taking expectation of the Eq. (32) we have the expression for MSE as:

33 $$\begin{aligned}\:MSE\left[{T}_{Ln\left(str\right)}\right]&={\bar{Y}}_{str}^{2}+{k}_{1}^{2}{\bar{Y}}_{str}^{2}+\frac{1}{4}{k}_{1}^{2}{\bar{Y}}_{str}^{2}{\nu}_{04}+{k}_{1}^{2}{\bar{Y}}_{str}^{2}{\nu}_{40}+{k}_{2}^{2}+\frac{1}{4}{k}_{2}^{2}{\nu}_{04}-2{k}_{1}{\bar{Y}}_{str}^{2}\\ & \quad+\frac{2}{3}{k}_{1}^{2}{\bar{Y}}_{str}^{2}{\nu}_{04}-{k}_{1}^{2}{\bar{Y}}_{str}^{2}{\nu}_{44}+{2k}_{1}{k}_{2}{\bar{Y}}_{str}+\frac{2}{3}{k}_{1}{k}_{2}{\bar{Y}}_{str}{\nu}_{04}-\frac{2}{3}{k}_{1}{\bar{Y}}_{str}^{2}{\nu}_{04} +{k}_{1}{\bar{Y}}_{str}^{2}{\nu}_{44}-2{k}_{2}{\bar{Y}}_{str}\\ & \quad-\:\frac{2}{3}{k}_{2}{\bar{Y}}_{str}{\nu}_{04}-\frac{1}{3}{k}_{1}^{2}{\bar{Y}}_{str}^{2}{\nu}_{04}-{k}_{1}^{2}{\bar{Y}}_{str}^{2}{\nu}_{44}+{k}_{1}{k}_{2}{\bar{Y}}_{str}{\nu}_{04}+\frac{2}{3}{k}_{1}{k}_{2}{\bar{Y}}_{str}{\nu}_{04}-{2k}_{1}{k}_{2}{\bar{Y}}_{str}{\nu}_{44}+\frac{2}{3}{k}_{2}^{2}{\nu}_{04}\end{aligned}$$

The equation has been simplified, and its simplified form is presented in Eq. (34) as follows.

34 $$\:MSE\left[{T}_{Ln\left(str\right)}\right]={k}_{1}^{2}{\bar{Y}}_{str}^{2}{A}_{11}-2{k}_{1}{\bar{Y}}_{str}^{2}{A}_{12}-2{k}_{1}{k}_{2}{\bar{Y}}_{str}{A}_{13}+{k}_{2}^{2}{A}_{14}-2{k}_{2}{\bar{Y}}_{str}{A}_{15}+{\bar{Y}}_{str}^{2}$$

Where

$$\:{A}_{11}={1+\nu}_{40}+\frac{11}{12}{\nu}_{04}-2{\nu}_{44}$$

$$\:{A}_{12}=1+\frac{11}{12}{\nu}_{04}$$

$$\:{A}_{13}=1+\frac{1}{3}{\nu}_{04}-{\nu}_{44}$$

$$\:{A}_{14}=1+\frac{1}{3}{\nu}_{04}$$

$$\:{A}_{15}=1+\frac{11}{12}{\nu}_{04}-{\nu}_{44}$$

The above Eq. (34) is differentiated with respect to $\:{k}_{1}$the following results are shown in Eq. (35) as:

35 $$\:-2{A}_{14}{\bar{Y}}_{str}^{2}{k}_{1}+2{A}_{12}{k}_{2}-2{\bar{Y}}_{str}{A}_{15}$$

And again the Eq. (34) is differentiated with respect to $\:{k}_{2}$ the following results are shown in Eq. (36) as:

36 $$\:-2{A}_{14}{\bar{Y}}_{str}^{2}{k}_{1}+2{A}_{12}{k}_{2}-2{\bar{Y}}_{str}{A}_{15}$$

To fine out the Optimum values of $\:MSE\left[{T}_{Ln\left(str\right)}\right]$, we differentiate it with respect to $\:{k}_{1}$ and $\:{k}_{2}$ by Calculus rule and equate to zero as follows.

$$\:\frac{\partial\:MSE\left[{T}_{Ln\left(str\right)}\right]}{\partial\:{k}_{1}}=2\:{A}_{11}{\bar{Y}}_{str}^{2}{k}_{1}-2{A}_{13}{\bar{Y}}_{str}^{2}-2{A}_{14}{\bar{Y}}_{str}{k}_{2}=0$$

$$\:\frac{\partial\:MSE\left[{T}_{Ln\left(str\right)}\right]}{\partial\:{k}_{2}}=\:-2{A}_{14}{\bar{Y}}_{str}^{2}{k}_{1}+2{A}_{12}{k}_{2}-2{\bar{Y}}_{str}{A}_{15}\:=0$$

Then we get the following expression,

$$\:\left[\begin{array}{cc}{\bar{Y}}_{str}^{2}{A}_{11}&\:-{\bar{Y}}_{str}{A}_{14}\\\:-{\bar{Y}}_{str}{A}_{14}&\:{A}_{13}\end{array}\right]\left[\begin{array}{c}{k}_{1}\\\:{k}_{2}\end{array}\right]=\left[\begin{array}{c}{\bar{Y}}_{str}^{2}{A}_{12}\\\:{\bar{Y}}_{str}{A}_{15}\end{array}\right]$$

By using the Cramer’s rules to solve the following equations:

37 $$\:\left\{{k}_{1\left(Optimum\right)}=\frac{{A}_{12}{A}_{13}-{A}_{14}{A}_{15}}{{A}_{11}{A}_{13}-{A}_{14}^{2}},{k}_{2\left(Optimum\right)}=\frac{{\bar{Y}}_{str}\left({A}_{11}{A}_{15}+{A}_{14}{A}_{12}\right)}{{A}_{11}{A}_{13}-{A}_{14}^{2}}\right\}$$

Again, put the value of $\:{k}_{1}and\:{k}_{2}$ in Eq. (34) then the following expressions can be written as:

38 $$\begin{aligned}&\frac{{A}{11}{\bar{Y}}{str}^{2}{\left({A}{12}{A}{13}+{A}{14}{A}{15}\right)}^{2}}{{\left({A}{11}{A}{13}-{A}{14}^{2}\right)}^{2}}-\frac{2{A}{12}{\bar{Y}}{str}^{2}\left({A}{12}{A}{13}+{A}{14}{A}{15}\right)}{{A}{11}{A}{13}-{A}{14}^{2}}-\frac{2{A}{13}{\bar{Y}}{str}^{2}\left({A}{12}{A}{13}+{A}{14}{A}{15}\right)\left({A}{11}{A}{14}+CE\right)}{{\left({A}{11}{A}{13}-{A}{14}^{2}\right)}^{2}}\\ & \quad +\frac{{A}{14}{\bar{Y}}{str}{\left({A}{11}{A}{14}+{A}{13}{A}{15}\right)}^{2}}{{\left({A}{11}{A}{13}-{A}{14}^{2}\right)}^{2}}-\frac{2{A}{15}{\bar{Y}}{str}\left({A}{11}{A}{14}+{A}{13}{A}{15}\right)}{{A}{11}{A}{13}-{A}{14}^{2}}+{\bar{Y}}{str}^{2}\end{aligned}$$

The simplified expression for the MSE of the proposed estimator is presented in Eq. (36) as following bellow:

39 $$\:MSE\left[{T}_{Ln\left(str\right)}\right]={\bar{Y}}_{str}^{2}\left(1-\frac{{A}_{11}{A}_{15}^{2}+{A}_{12}^{2}{A}_{13}-2{A}_{13}{A}_{14}{A}_{15}}{{A}_{11}{A}_{13}-{A}_{14}^{2}}\right)$$

## Efficiency comparison

To assess the criteria under which the proposed estimators achieve higher efficiency than existing alternatives. The superior performance of the estimators is contingent upon the satisfaction of specific mathematical criteria. These include lower bias and reduced MSE and Percentage Relative Efficiency (PRE) to traditional estimators. Only when these criteria are met can the proposed methods be considered more effective over the competing estimators.

**Condition 1**:

Comparing Eq. (39) and Eq. (2), the proposed estimator will outperform the traditional unbiased estimator if:

$$\:MSE\left({T}_{Ln\left(str\right)}\right)<\:MSE\left({T}_{1}\right)$$

i.e.

40 $$\:MSE\left[{T}_{Ln\left(str\right)}\right]={\bar{Y}}_{str}^{2}\left(1-\frac{{A}_{11}{A}_{15}^{2}+{A}_{12}^{2}{A}_{13}-2{A}_{13}{A}_{14}{A}_{15}}{{A}_{11}{A}_{13}-{A}_{14}^{2}}\right)$$

where

$$\:\varOmega\:=\:{\bar{Y}}_{str}^{2}\left(1-\frac{{A}_{11}{A}_{15}^{2}+{A}_{12}^{2}{A}_{13}-2{A}_{13}{A}_{14}{A}_{15}}{{A}_{11}{A}_{13}-{A}_{14}^{2}}\right)$$

**Condition 2**:

By comparing Eq. (39) and Eq. (5), the proposed estimator will outperform the Cochran^10^ estimator if:

$$\:MSE\left({T}_{Ln\left(str\right)}\right)<\:MSE\left({T}_{2}\right)$$

i.e.

41 $$\:\left[\left({\mathrm{V}}_{40}+{\mathrm{V}}_{04}-2{\mathrm{V}}_{44}-1+\varOmega\:\right)\right]>0$$

**Condition 3**:

By comparing of Eq. (39) and Eq. (8), the proposed estimator will outperform the Bahl et al.^11^ estimator if:

$$\:MSE\left({T}_{Ln\left(str\right)}\right)<\:MSE\left({T}_{3}\right)$$

i.e.

42 $$\:\left[{V}_{40}+\frac{1}{4}{V}_{04}-{2V}_{44}\right]-1+\:\varOmega\:>0$$

**Condition 4**:

By comparing of Eq. (39) and Eq. (11), the proposed estimator will outperform T_4_ estimator if:

$$\:MSE\left({T}_{Ln\left(str\right)}\right)<\:MSE\left({T}_{4}\right)$$

43 $$\:\left[{\nu}_{40}+{\vartheta\:}^{2}{\nu}_{04}-2\vartheta\:{\nu}_{44}\right]-1+\:\varOmega\:>0$$

**Condition 5**:

By comparing of Eq. (39) and Eq. (14), the proposed estimator will outperform the Upadhyaya et al.^14^ estimator if:

$$\:MSE\left({T}_{Ln\left(str\right)}\right)<\:MSE\left({T}_{5}\right)$$

i.e.

44 $$\:\left[{\nu}_{40}+{\phi\:}^{2}{\nu}_{04}-2\phi\:{\nu}_{44}\right]-1+\:\varOmega\:>0$$

**Condition 6**:

By comparing of Eq. (39) and Eq. (17), the proposed estimator will outperform the Kadilar and Cingi^16^ estimator if:

$$\:MSE\left({T}_{Ln\left(str\right)}\right)<\:MSE\left({T}_{6}\right)$$

i.e.

45 $$\:\left[{\nu}_{40}+{\alpha\:}^{2}{\tau\:}^{2}{\theta\:}^{2}{\nu}_{04}-2\alpha\:\tau\:\theta\:{\nu}_{44}\right]-1+\:\varOmega\:>0$$

**Condition 7**:

By comparing of Eq. (39) and Eq. (20), the proposed estimator will outperform the Onyeka [22] estimator if:

$$\:MSE\left({T}_{Ln\left(str\right)}\right)<\:{MSE(T}_{7})$$

i.e.

46 $$\:\frac{{\nu}_{01}{\nu}_{02}-{\nu}_{22}^{2}}{{\nu}_{01}+{\nu}_{02}-2{\nu}_{22}}-{\bar{Y}}^{2}\left[1-\:\varOmega\:\right]>0$$

**Condition 8**:

By comparing of Eq. (3) and Eq. (24), the proposed estimator will outperform the Izunobi and Onyeka^19^ estimator if:

$$\:MSE\left({T}_{Ln\left(str\right)}\right)<\:MSE\left({T}_{8}\right)$$

i.e.

47 $$\:\left[{\nu}_{40}\left(1-{\rho}_{t}\right)\right]-1+\:\varOmega\:>0$$

The statements presented above serve as the foundation for establishing the circumstances in which the newly proposed estimator’s exhibit improved performance over existing methods. When these specified conditions are satisfied, the effectiveness and innovation of the proposed estimators are validated. In essence, these criteria are directly linked to the efficiency and practical advantage of the new estimators.

## Real life datasets

To assess the practical performance of the proposed estimators, we apply them to real-life datasets. These datasets reflect the variability and structure typically encountered in survey sampling, allowing for a meaningful evaluation of estimator efficiency and accuracy. The results provide insights into the applicability and advantages of the proposed methods in real-world settings.

**Data-I: Source: Kadilar and Cingi**^16^.

Data Set-I Kadilar and Cingi^16^, provides information on apple yields (Y) along with the corresponding number of apple trees (X) in each village. For analytical purposes, the villages are organized into strata based on their geographical regions within the country.

**Data-II: Source: Daraz and Shabbir**^20^.

Data Set-II, Daraz and Shabbir^20^, contains 2012 statistics organized by administrative divisions. It includes information on student enrollment figures (Y) and the number of government schools (X) within each division.

**Data-III: Source**: Kadilar and Cingi^21^.

Data Set-III, Kadilar and Cingi^21^, focuses on the number of wet days as the primary variable (Y), with total sunshine hours serving as the supporting (X) variable.

Table 1 presents the summary statistics of the datasets described above.

**Table 1:** Summary Statistics for All Datasets.

Data	Stratum	$\:{\boldsymbol{N}}_{\boldsymbol{h}}$	$\:{\boldsymbol{n}}_{\boldsymbol{h}}$	$\:{\bar{\boldsymbol{Y}}}_{\boldsymbol{h}}$	$\:{\bar{\boldsymbol{X}}}_{\boldsymbol{h}}$	$\:{\boldsymbol{S}}_{\boldsymbol{y}\boldsymbol{h}}$	$\:{\boldsymbol{S}}_{\boldsymbol{x}\boldsymbol{h}}$	$\:{\boldsymbol{\rho\:}}_{\boldsymbol{y}\boldsymbol{x}\boldsymbol{h}}$	$\:{\boldsymbol{C}}_{\boldsymbol{y}\boldsymbol{h}}$	$\:{\boldsymbol{C}}_{\boldsymbol{x}\boldsymbol{h}}$
I	i	10	4	149.7	1630	102.17	13.470	-0.779	0.063	0.09
	ii	10	4	102.6	2036	103.26	12.610	-0.503	0.050	0.122
II	i	18	8	1979.3	962.05	2587.7	307.953	0.1447	1.570	0.320
	ii	18	8	134,458	1146.7	50235.82	469.931	0.787	0.373	0.4098
III	i	106	9	1536	127	49,189	6425	0.82	4.18	2.02
	ii	106	17	2212	117	57,461	11,552	0.86	5.22	2.1
	iii	94	38	9384	103	160,757	29,909	0.9	3.19	2.22
	iv	171	67	5588	170	285,603	28,643	0.99	5.13	3.84
	v	204	7	967	205	45,403	2390	0.71	2.47	1.75
	vi	173	2	404	201	18,794	946	0.89	2.34	1.91

Table 2 reports the MSE of both conventional and proposed estimators under SRS with a single secondary variable. The first two populations are divided into two strata each, while the third population is partitioned into six strata. Among the proposed estimators, $\:{T}_{Ln\left(str\right)}$ is a nonlinear estimator that incorporates auxiliary information from the related variable X to estimate the population mean of Y. It utilizes the known population mean of X and applies a logarithmic adjustment based on the ratio $\:Ln\left(\frac{{\bar{x}}_{str}}{{\mu}_{xstr}}\right)$, modifying the estimate of Y accordingly. This estimator aims to improve precision and reliability by minimizing bias and MSE, especially when there is a strong correlation between the study and auxiliary variables. The comparative results confirm the superior performance of the proposed estimators, as reflected in their lower MSE values (e.g., 7.56 × 10⁻¹, 2.49 × 10^7^, and 7.48 × 10^4^) compared to all existing estimators.

**Table 2:** Empirical Evaluation of Proposed Estimator Performance Using Real Datasets.

Estimators	Data-I	Data-II	Data-III
$\:{T}_{1}$	8.87 × 10^0^	1.18 × 10^9^	6.74 × 10⁵
$\:{T}_{2}$	1.8795 × 10¹	1.13 × 10^9^	1.59 × 10⁵
$\:{T}_{3}$	1.3080 × 10¹	1.13 × 10^9^	3.41 × 10⁵
$\:{T}_{4}$	1.8795 × 10¹	1.13 × 10^9^	1.59 × 10⁵
$\:{T}_{5}$	1.8795 × 10¹	1.13 × 10^9^	1.59 × 10^9^
$\:{T}_{6}$	1.29574 × 10³	4.52 × 10^9^	9.98 × 10⁶
$\:{T}_{7}$	4.945 × 10^0^	1.12 × 10^9^	1.07 × 10⁵
$\:{T}_{8}$	4.945 × 10^0^	1.12 × 10^9^	1.22 × 10⁵
$\:{\boldsymbol{T}}_{\boldsymbol{L}\boldsymbol{n}\left(\boldsymbol{s}\boldsymbol{t}\boldsymbol{r}\right)}$	7.56 × 10⁻¹	2.49 × 10^7^	7.48 × 10⁴

Furthermore, Table 3 proposed estimators exhibit higher Percent Relative Efficiency (PRE) values 186.82, 438.48, and 900.867 indicating a significant gain in estimation accuracy across various population structures and stratified sampling scenarios.

**Table 3:** Percentage Relative Efficiencies of Estimators Using Real Data.

Estimators	Data-I	Data-II	Data-III
	*n* = 50	*n* = 100	*n* = 150
$\:{T}_{1}$	24.45	36.37	42.47
$\:{T}_{2}$	24.24	41.75	47.83
$\:{T}_{3}$	67.87	83.45	93.56
$\:{T}_{4}$	47.25	79.74	87.57
$\:{T}_{5}$	50.23	75.75	94.52
$\:{T}_{6}$	62.55	79.45	85.08
$\:{T}_{7}$	64.57	104.50	129.62
$\:{T}_{8}$	179.25	204.51	459.41
$\:{\boldsymbol{T}}_{\boldsymbol{L}\boldsymbol{n}\left(\boldsymbol{s}\boldsymbol{t}\boldsymbol{r}\right)}$	**186.82**	**438.48**	**712.86**

## Simulation study

**Fig. 1:**
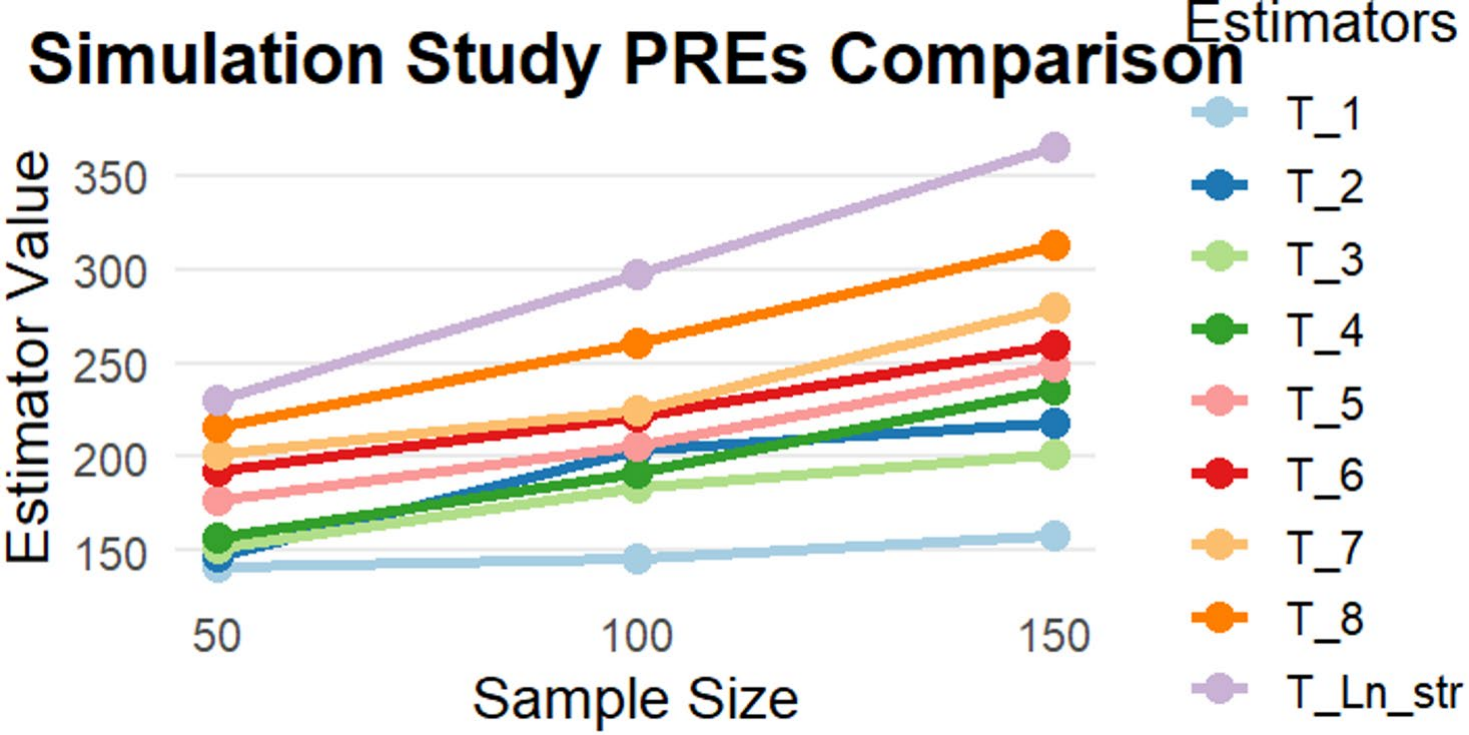
PREs of Proposed Estimators and Existing Estimators Using Simulations Datasets.

A finite population was generated in which the study variable was assumed to have a strong association with an auxiliary variable, reflecting practical survey conditions. Stratified random sampling without replacement was adopted, and samples of sizes *n* = 50, 100, and 150 were drawn using proportional allocation across strata. For each sampled dataset, all competing estimators, including the proposed logarithmic estimator, were computed and compared. This process was repeated 1,0000 times to account for Monte Carlo variability. The performance of the estimators was evaluated using the PRE, calculated with respect to a baseline estimator, in order to assess the relative gain or loss in efficiency of the proposed estimator under varying sample sizes as presented in Table 4; Fig. 1.

**Table 4:** Simulation-Based Comparison of Percentage Relative Efficiencies of Estimators.

Estimators	Data-I	Data-II	Data-III
	*n* = 50	*n* = 100	*n* = 150
$\:{T}_{1}$	141.34	145.65	158.35
$\:{T}_{2}$	147.23	203.75	218.52
$\:{T}_{3}$	151.87	184.08	201.66
$\:{T}_{4}$	157.23	191.75	236.48
$\:{T}_{5}$	177.23	205.75	248.52
$\:{T}_{6}$	192.34	221.40	259.49
$\:{T}_{7}$	201.50	224.50	279.62
$\:{T}_{8}$	216.25	260.50	311.419
$\:{\boldsymbol{T}}_{\boldsymbol{L}\boldsymbol{n}\left(\boldsymbol{s}\boldsymbol{t}\boldsymbol{r}\right)}$	231.02	297.48	364.91

**Fig. 2:**
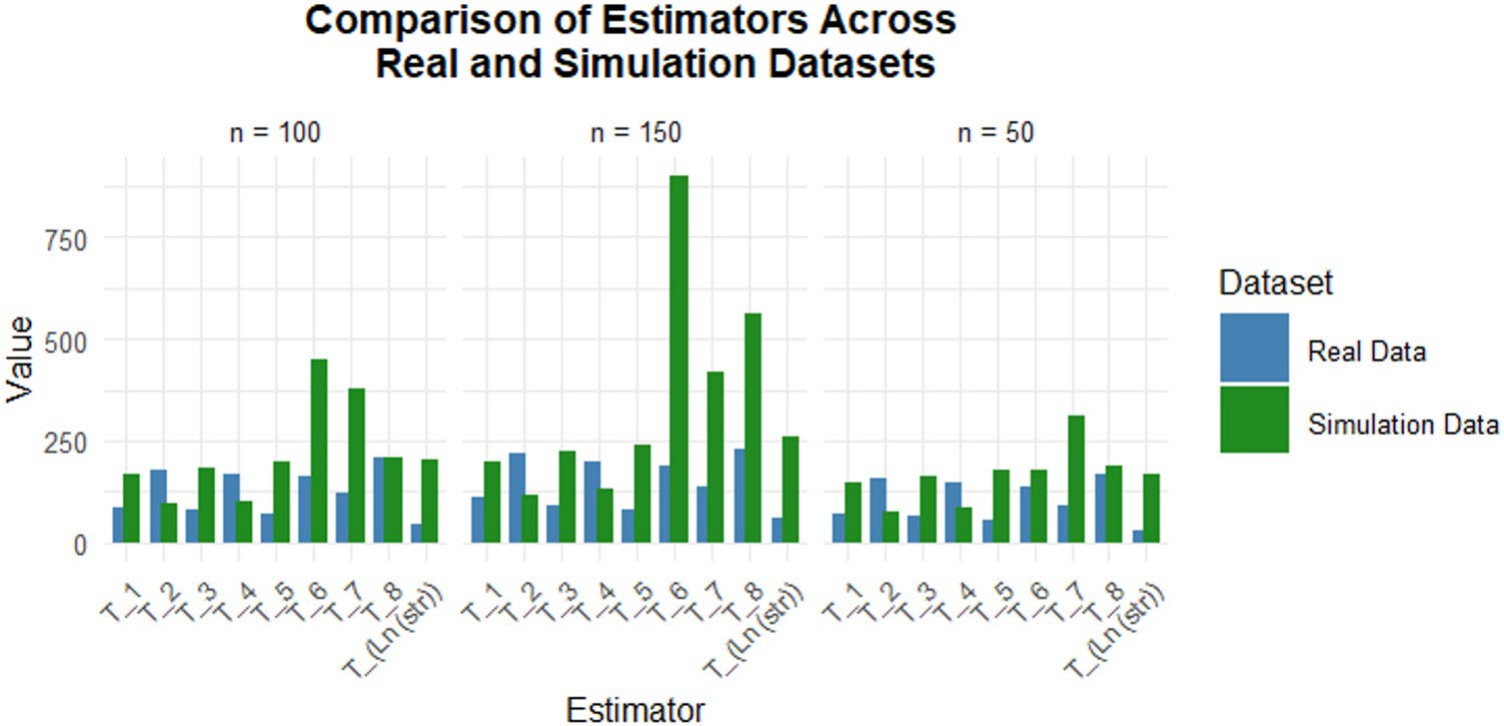
Comparing Proposed and Existing Estimators using Real and Simulated Datasets.

The Fig. 2 illustrates a comparative performance of different estimators using both real and simulated datasets across varying sample sizes $\:\left(n=50,\:100,\:and\:150\right)$ Overall, estimator efficiency improves as the sample size increases, with clearer performance differentiation at larger n. The proposed logarithmic-type estimator consistently demonstrates substantially higher efficiency values compared to the competing estimators. The widening performance gap at larger samples further highlights the robustness, consistency, and practical reliability of the proposed estimator under diverse sampling scenarios.

## Discussion

In this study, we proposed a novel class of logarithmic-type estimators within the framework of stratified random sampling, leveraging information from a single auxiliary variable to improve the estimation of the population mean. The theoretical development included derivations of bias and MSE using first-order approximations, providing a solid foundation for assessing the statistical properties and efficiency of the proposed estimators. To evaluate their performance relative to traditional methods, we established criteria for minimizing MSE and enhancing estimation accuracy. The proposed estimators were designed to outperform existing approaches under conditions that optimize the use of auxiliary information. Both real-world datasets and simulation studies were employed to assess practical applicability and robustness across diverse population structures. The empirical results demonstrate that the proposed estimators consistently achieve lower MSEs and higher percentage relative efficiencies compared to standard stratified mean, ratio, and regression estimators. These findings indicate improved precision in estimating the population mean and reflect the estimators’ ability to effectively exploit the correlation between the study and auxiliary variables. Moreover, the proposed estimators exhibit reduced variability in performance across different sample sizes, suggesting stable and reliable behavior regardless of sampling fluctuations.

Further analysis reveals that the efficiency gains are particularly pronounced when strong correlations exist between the study and auxiliary variables. In scenarios where multiple strata exhibit high correlation, the proposed estimators achieve even greater improvements, highlighting their adaptability across varied correlation structures. These results demonstrate that the logarithmic transformation enhances both the precision and reliability of the estimators, making them suitable for a wide range of practical applications.

## Conclusion

A new logarithmic-type estimator is proposed for estimating the population mean within the stratified random sampling framework using one auxiliary variable. The bias and MSE of the estimator are obtained using first-order approximations. To assess its comparative advantage, specific conditions are established under which the proposed estimator outperforms conventional methods. An extensive empirical and simulation-based evaluation is conducted using multiple real-life datasets and 1000 simulated samples generated from a normal population, in which the MSE and PRE of the projected estimator are systematically linked with those of traditional estimators. The consistency of the results across different datasets highlights the practical reliability and enhanced precision of the proposed estimator, thereby confirming its overall superiority across diverse sampling scenarios.

This research highlights the significant contribution of the proposed logarithmic-type estimator developed for stratified random sampling with a one supplementary variable. By incorporating a logarithmic conversion, the estimator more effectively captures the association among the study and supplementary variables, resulting in enhanced estimation accuracy. Both theoretic investigation and empirical evaluation demonstrate that the estimator achieves higher precision, reliability, and robustness across diverse population distributions, confirming its practical usefulness.

In conclusion, the proposed logarithmic-type estimator provides a robust framework for improving population mean estimation in complex sampling structures. Future research may extend this approach to settings involving multiple auxiliary variables and examine its performance under alternative sampling designs. Additionally, the methodology offers potential for addressing practical challenges such as nonresponse and for enhancing estimator efficiency under diverse distributional conditions.

## Supplementary Information

Below is the link to the electronic supplementary material.

Supplementary Material 1

## Data Availability

All data generated or analyzed during this study are included in this published article.
