# Supplementary material for: An efficient logarithmic estimator in stratified random sampling using single auxiliary variable
Source: Sci Rep. 2026 Feb 26;16:11126. doi: 10.1038/s41598-026-41448-9 (PMC13047051; doi:10.1038/s41598-026-41448-9)
Supplement: Supplementary file 1 — Supplementary Material 1 [file 41598_2026_41448_MOESM1_ESM.pdf]

R codes for the Manuscript Title "An Efficient Logarithmic Estimator in Stratified Random Sampling Using Single Auxiliary Variable"

PREs values on Real Data sets

```
# Load libraries

library(ggplot2)

library(tidyr)

library(dplyr)


# -----------------------

# Real Data

# -----------------------

real_data <- data.frame(

  Estimator = c("T_1","T_2","T_3","T_4","T_5","T_6","T_7","T_8","T_Ln_str"),

  n_50   = c(24.45, 24.24, 67.87, 47.25, 50.23, 62.55, 64.57, 179.25, 186.82),

  n_100 = c(36.37, 41.75, 83.45, 79.74, 75.75, 79.45, 104.50, 204.51, 438.48),

  n_150 = c(42.47, 47.83, 93.56, 87.57, 94.52, 85.08, 129.62, 459.41, 712.86)

)


# -----------------------

# Convert wide → long

# -----------------------

real_long <- real_data %>%

  pivot_longer(

    cols = starts_with("n_"),

    names_to = "SampleSize",
```

```
    values_to = "PRE"
  ) %>%
  mutate(
    SampleSize = factor(
      SampleSize,
      levels = c("n_50","n_100","n_150"),
      labels = c("50","100","150")
    )
  )

# -----------------------
# Fixed colors for estimators
# -----------------------
estimator_colors <- c(
  "T_1"         = "#a6cee3",
  "T_2"         = "#1f78b4",
  "T_3"         = "#b2df8a",
  "T_4"         = "#33a02c",
  "T_5"         = "#fb9a99",
  "T_6"         = "#e31a1c",
  "T_7"         = "#fdbf6f",
  "T_8"         = "#ff7f00",
  "T_Ln_str"   = "#cab2d6"
)
```

```
# ----------------------
# Line plot
# ----------------------
ggplot(real_long,
       aes(x = SampleSize, y = PRE,
           group = Estimator, color = Estimator)) +
  geom_line(size = 1.2) +
  geom_point(size = 3) +
  scale_color_manual(values = estimator_colors) +
  theme_minimal(base_size = 12) +
  theme(
    plot.title = element_text(face = "bold", size = 16, hjust = 0.5),
    axis.title = element_text(face = "bold"),
    legend.title = element_text(face = "bold"),
    legend.position = "right"
  ) +
  labs(
    title = "PREs Values on Real Data",
    x = "Sample Size",
    y = "Estimator Value",
    color = "Estimators"
  )
```

PRS values on Simulation data

```
# Load libraries
library(ggplot2)
```

```
library(tidyr)

library(dplyr)


# -----------------------

# New Data

# -----------------------

real_data <- data.frame(

  Estimator = c("T_1","T_2","T_3","T_4","T_5","T_6","T_7","T_8","T_Ln_str"),

  n_50   = c(141.34, 147.23, 151.87, 157.23, 177.23, 192.34, 201.50, 216.25, 231.02),

  n_100 = c(145.65, 203.75, 184.08, 191.75, 205.75, 221.40, 224.50, 260.50, 297.48),

  n_150 = c(158.35, 218.52, 201.66, 236.48, 248.52, 259.49, 279.62, 311.419, 364.91)

)


# -----------------------

# Convert wide → long

# -----------------------

real_long <- real_data %>%

  pivot_longer(

    cols = starts_with("n_"),

    names_to = "SampleSize",

    values_to = "PRE"

  ) %>%

  mutate(

    SampleSize = factor(

      SampleSize,
```

```
        levels = c("n_50","n_100","n_150"),
        labels = c("50","100","150")
      )
  )

# -----------------------
# Fixed colors for estimators
# -----------------------
estimator_colors <- c(
  "T_1"      = "#a6cee3",
  "T_2"      = "#1f78b4",
  "T_3"      = "#b2df8a",
  "T_4"      = "#33a02c",
  "T_5"      = "#fb9a99",
  "T_6"      = "#e31a1c",
  "T_7"      = "#fdbf6f",
  "T_8"      = "#ff7f00",
  "T_Ln_str" = "#cab2d6"
)

# -----------------------
# Line plot
# -----------------------
ggplot(real_long,
         aes(x = SampleSize, y = PRE,
```

```
                group = Estimator, color = Estimator)) +
  geom_line(size = 1.2) +
  geom_point(size = 3) +
  scale_color_manual(values = estimator_colors) +
  theme_minimal(base_size = 12) +
  theme(
    plot.title = element_text(face = "bold", size = 16, hjust = 0.5),
    axis.title = element_text(face = "bold"),
    legend.title = element_text(face = "bold"),
    legend.position = "right"
  ) +
  labs(
    title = "Simulation Study PREs Comparison",
    x = "Sample Size",
    y = "Estimator Value",
    color = "Estimators"
  )
```

Comaprison of Estimators on Real and Simulation data sets

```
# Load libraries
library(ggplot2)
library(dplyr)
library(tidyr)

# -----------------------
```

```
# Real Data
# ----------------------
real_data <- data.frame(
  Estimator = c("T_1","T_2","T_3","T_4","T_5","T_6","T_7","T_8","T_Ln(str)"),
  n_50    = c(24.45, 24.24, 67.87, 47.25, 50.23, 62.55, 64.57, 179.25, 186.82),
  n_100 = c(36.37, 41.75, 83.45, 79.74, 75.75, 79.45,104.50, 204.51, 438.48),
  n_150 = c(42.47, 47.83, 93.56, 87.57, 94.52, 85.08,129.62, 459.41, 712.86)
)

real_data$Dataset <- "Real Data"

# ----------------------
# Simulation Data
# ----------------------
simulation_data <- data.frame(
  Estimator = c("T_1","T_2","T_3","T_4","T_5","T_6","T_7","T_8","T_Ln(str)"),
  n_50    = c(141.34,147.23,151.87,157.23,177.23,192.34,201.50,216.25,231.02),
  n_100 = c(145.65,203.75,184.08,191.75,205.75,221.40,224.50,260.50,297.48),
  n_150 = c(158.35,218.52,201.66,236.48,248.52,259.49,279.62,213.42,364.91)
)

simulation_data$Dataset <- "Simulation Data"

# ----------------------
# Combine datasets (Real FIRST)
```

```r
# -----------------------
combined <- bind_rows(real_data, simulation_data)


# -----------------------
# Convert wide → long format
# -----------------------
combined_long <- combined %>%
  pivot_longer(
    cols = starts_with("n_"),
    names_to = "SampleSize",
    values_to = "Value"
  ) %>%
  mutate(
    SampleSize = factor(
      SampleSize,
      levels = c("n_50","n_150","n_100"),
      labels = c("n = 50","n = 150","n = 100")
    ),
    Dataset = factor(Dataset, levels = c("Real Data","Simulation Data"))
  )


# -----------------------
# Grouped Bar Chart
# -----------------------
ggplot(combined_long, aes(x = Estimator, y = Value, fill = Dataset)) +
  geom_bar(stat = "identity", position = position_dodge(width = 0.8), width = 0.7) +
```

```
facet_wrap(~SampleSize, nrow = 1) +
scale_fill_manual(values = c("Real Data" = "#1f78b4", "Simulation Data" = "#33a02c")) +
theme_minimal(base_size = 9) +
theme(
  axis.text.x = element_text(angle = 45, hjust = 1),
  strip.background = element_rect(fill = "lightgray", color = NA),
  legend.position = "right"
) +
labs(
  title = "Comparison of Estimators Across
  Real and Simulation Datasets",
  x = "Estimator",
  y = "Value",
  fill = "Dataset"
)
```
